# Kansas City

**Published:** 1896-08

**Authors:** 


Kansas City Veterinary College.—No announcement for the coming collegiate year promises so much for the future veterinary student in extended changes of curriculum as that of the Kansas City Veterinary College. No more gratifying news could be afforded the profession and its associations than this marked progress in behalf of higher veterinary education. The sincerity of purpose of those who have directed this school for the past three years has been of a character to establish confidence in the minds of all, and we need no better evidence of the intentions and purposes of those who will direct and control it in the future than the announcement just issued for the course of 1896–97.

Entering a new home, where greater room and better facilities for work greet them, and adopting a fixed course of instruction over a period of three years and six months each, they will surely find that they have not built in vain, for the strength and power of any school is not in the claim of a few shining lights among the profession, who represent the school as graduates, but rather in the uniform strength of all their offspring and the recognition and support they command as a whole because of their worth and merit.

The complete reorganization of the faculty has afforded an opportunity of assigning the work in a more complete manner and the readjusting of the course of instruction in thorough accord with the needs of the times.

Dr. Charles J. Sihler adds Anatomy to his other branch of Meat-inspection, while Clinical Surgery has been placed under the direction of Robert C. Moore, D.V.S., vice John Forbes, M.R.C.V.S., who will not be connected with the school this year.

The Control and Eradication of Contagious Diseases has been placed under the direction of Dr. T. J. Turner, late State Veterinarian of Missouri, ex-Secretary of the United States Veterinary Medical Association, a graduate of the American Veterinary College, vice George C. Pritchard, V.S., resigned.

The subject of Principles and Practice of Medicine, Milk- and Dairy-inspection remain under Dr. Sesco Stewart, and the same may be said of Materia Medica and Therapeutics, which will be taught by Dr. S. L. Hunter.

Dr. Silas L. Brooking will adhere to the teaching of Comparative Physiology, while the former branch of Anatomy connected with this chair goes to Frederick Hopkins, D.V.S., who has been added to the faculty, assisted by L. Enos Day, V.S., who succeeds John E. Topping, D.V.S., who held the position of Demonstrator of Anatomy in the session of 1895–96.

Thomas A. Bray, D.V.S., has been added to the teaching staff, and will instruct the students in Lameness and Shoeing and Physical Diagnosis. These branches were formerly taught by Dr. Junius H. Wattles, whose health compelled him to resign his connection with the school (whose course he largely moulded), and for a time seek a milder climate.

Benjamin F. Kaupp, D.V.S., succeeds John Airth, M.R.C.V.S., resigned, as instructor on Parasites and Parasitic Diseases.

R. H. Harrison, D.V.S., retains the subjects of Canine Pathology and Dental Surgery as his work. Likewise William C. Barth, V.S., that of Obstetrics.

Theodore Schaefer, M.D., succeeds Claude C. Hamilton, M.D., Ph. G., as Instructor in Chemistry, and Joseph S. Lichtenberg, M.D., succeeds Flavell B. Tiffany, M.D., on the subject of Ophthalmology. George Q. Smith will aid the Chair of Chemistry as demonstrator.

Hygiene has been added to the list of subjects taught, and will be directed by E. Von Mast, M.D.

U. B. McCurdy, D.V.S., succeeds John E. Topping, D.V.S., on Breeds and Breeding and Feeding of Animals.

Veterinary Jurisprudence has been added to the course and assigned to Frank M. Sheridan, Esq.

Isidore J. Wolf, M.D., will consider the subject of Bacteriology, succeeding Leo. A. Schaeffer, M.D., and Nathan McVey, M.D., resigned. His former subjects of Microscopy and Histology will be taught by Leon Rosenwald, M.D., under the heads of Histology and Pathology, assisted by Thomas C. Procter, M.D., as demonstrator.

O. G. Atherton, D.V.S., has been added to the faculty as Demonstrator of Materia Medica and Pharmacy.

The total corps of instructors number twenty-two, an increase of four over the preceding session; of this number fourteen are practising veterinarians while six are practising human physicians, one a member of the bar, and another giving his attention to laboratory work.

Beginning in January of each year a post-graduate course will be inaugurated, lasting eight weeks, covering specially the subjects of Meat-inspection, Dairy- and Milk-inspection.

The entrance examination established by the Association of Veterinary Faculties will be adhered to, in the absence of certificate or diploma from some other college, normal, or high school. One hundred dollars for tuition fee for each year has been established, covering all expenses, and a fee of fifty dollars for the post graduate course.

---

### WHAT WE WANT TO KNOW.

How those people who are the promoters of horse-shows expect to improve the type and character of the horses in the sections of the country where they are held, when no proper consideration is given to the relative soundness of the animals exhibited? Without a skilled, a trained veterinarian to conduct this all-important feature, there can be little expected in this direction.

How so many veterinarians in New York and Kings Counties were allowed to register by affidavit?

